# Association of In-Lab and Free-Living Mobility Measures With Falls and Fear of Falling in Underactive Older People

**DOI:** 10.1093/geroni/igab046.1730

**Published:** 2021-12-17

**Authors:** Stacey Schepens Niemiec, Cheryl Vigen, Jeanine Blanchard, Matthew Niemiec, Brittany Eng

**Affiliations:** 1 University of Southern California, Los Angeles, California, United States; 2 Independent contractor, Glendale, California, United States

## Abstract

Falls in older adults have significant consequences—a single fall can lead to serious injury, psychological trauma, activity restriction, and increased mortality. This study describes differences in mobility-related characteristics of underactive (<150 minutes/week of physical activity), racially diverse, older adults (65–84 years) classified by self-reported fall status (0, 1, 2+ falls in previous 12mo) and fear of falling (yes/no). We analyzed baseline data from 105 individuals (mean age=72.1 years; 73% female; 64% white, 29% Black, 12% Asian) who participated in a trial of a physical activity smartphone intervention for older people. Total minutes of daily stepping and medium-to-brisk (≥75 steps/min) and brisk (≥100) cadence bouts in free-living conditions was gathered over 3 days via ActivPal activity monitor. Gait speed was determined from a 4-meter walk test for those pretested prior to COVID-19 mandates (n=60). Of the median 81.8 minutes spent stepping daily, very few minutes involved moderate-to-brisk (14.0) or brisk cadence (10.1). Groups classified by fall status (non-fallers n=74, 1x fallers n=18, repeat fallers n=13) differed significantly in daily minutes spent in medium-to-brisk (p=0.04) and brisk cadence (p=0.02), but not in 4-meter gait speed or total minutes stepping. Individuals who reported fear of falling versus those with no fear did not differ significantly on any mobility-related parameters. Four-meter gait speed was significantly negatively correlated with both cadence measures (p=0.02) but not total minutes stepping. This study indicates that faster walking behaviors in everyday activity may be a useful target for intervention to prevent falls in underactive older adults.

